# Do allopatric male *Calopteryx virgo* damselflies learn species recognition?

**DOI:** 10.1002/ece3.90

**Published:** 2012-03

**Authors:** Katja Kuitunen, Elina Haukilehto, Kaisa J Raatikainen, Hanne Hakkarainen, Minna Miettinen, Harri Högmander, Janne S Kotiaho

**Affiliations:** 1 Department of Biological and Environmental Science, Centre of Excellence in Evolutionary Research, University of Jyväskylä,P.O. Box 35, FIN-40014 Jyväskylä, Finland.; 2 Nature and Resources, Nature and Cultural Environment Division, Centre for Economic Development, Transport and the Environment for Central Finland,P.O. Box 250, FIN-40101 Jyväskylä, Finland.; 3 Department of Mathematics and Statistics, University of Jyväskylä,P.O. Box 35, FIN-40014 Jyväskylä, Finland.; 4 Department of Mathematics and Statistics, University of Helsinki,P.O. Box 68, FIN-00014 Helsinki, Finland.; 5 Natural History Museum, University of Jyväskylä,Finland.

**Keywords:** Odonata, premating reproductive isolation, reinforcement, speciation, species discrimination

## Abstract

There is a growing amount of empirical evidence that premating reproductive isolation of two closely related species can be reinforced by natural selection arising from avoidance of maladaptive hybridization. However, as an alternative for this popular reinforcement theory, it has been suggested that learning to prefer conspecifics or to discriminate heterospecifics could cause a similar pattern of reinforced premating isolation, but this possibility is much less studied. Here, we report results of a field experiment in which we examined (i) whether allopatric *Calopteryx virgo* damselfly males that have not encountered heterospecific females of the congener *C. splendens* initially show discrimination, and (ii) whether *C. virgo* males learn to discriminate heterospecifics or learn to associate with conspecifics during repeated experimental presentation of females. Our experiment revealed that there was a statistically nonsignificant tendency for *C. virgo* males to show initial discrimination against heterospecific females but because we did not use sexually naïve individuals in our experiment, we were not able to separate the effect of innate or associative learning. More importantly, however, our study revealed that species discrimination might be further strengthened by learning, especially so that *C. virgo* males increase their association with conspecific females during repeated presentation trials. The role of learning to discriminate *C. splendens* females was less clear. We conclude that learning might play a role in species recognition also when individuals are not naïve but have already encountered potential conspecific mates.

## Introduction

It is generally well understood that some restriction to gene flow between populations is necessary for speciation to arise, and that under such restricted gene flow, populations can start to diverge and finally form new species (e.g., Coyne and Orr 2004; Rundle and Nosil 2005; Fitzpatrick et al. 2008). What has been the focus of much of the current speciation research are the mechanisms that keep the populations from mixing in cases where reproductive barriers are not yet fully developed (e.g., Coyne and Orr 2004). When reproductive isolation is not complete, interspecific matings and hybridization can lead into negative fitness consequences for the parental individuals thus increasing variation in fitness among the individuals of the populations. Such variation in fitness will cause natural selection against hybridization and thus may lead into adaptations to enhance the discrimination ability and as a consequence, reinforce premating reproductive isolation between the individuals of the interacting populations (e.g., Dobzhansky 1951; Howard 1993; Sætre et al. 1997; Higgie et al. 2000; Servedio 2001; Servedio and Noor 2003; Lukhtanov et al. 2005).

There are alternatives for the reinforcement theory, which may cause a similar pattern to the premating reproductive isolation. One of these alternatives is learning: learning related to species recognition has been documented in several animal taxa such as birds (Clayton 1989; Irwin and Price 1999; Price 2007), fish (Magurran and Ramnarine 2004; Verzijden and ten Gate 2007), and insects (Dukas 2004, 2009; Mery et al. 2009; Svensson et al. 2010). However, the evolutionary importance of learning in the species recognition context is still not widely known. Moreover, the little empirical evidence that there exists is conflicting: some studies find support for the role of learning in species recognition (Dukas 2004, 2009; Magurran and Ramnarine 2004; Verzijden and ten Cate 2007; Svensson et al. 2010) while other do not (Kandul et al. 2006).

Learning in the context of species recognition can occur in two ways: individuals can either learn to discriminate heterospecifics individuals (hereafter discrimination learning), and/or learn to prefer conspecifics (hereafter association learning) (seePrice 2007). The former needs the presence of heterospecifics because individuals learn to discriminate through interactions with heterospecifics, while the latter does not. The latter type of learning occurs when animals learn to recognize conspecific individuals through imprinting during their juvenile or immature period, or through experience when adults. It has been suggested that imprinting to parents causes offspring to select mates of the type of their parents later in their life (reviewed in:Clayton 1989; Irwin and Price 1999; Moore 2004). In fact, it seems that at least in some circumstances, premating reproductive isolation may be reinforced more strongly through learning via sexual imprinting than through adaptation (Servedio et al. 2009). However, in species where parental care does not exist, imprinting is not possible, and individuals can only learn to discriminate or to associate with potential mates through experience (Irwin and Price 1999; Magurran and Ramnarine 2004). Imprinting and learning via experience are not mutually exclusive: in species with sexual imprinting, further learning can take place through experience later in life (Clayton 1989; Irwin and Price 1999; Price 2007). In addition, discrimination of heterospecifics or preferences for conspecifics can ultimately be genetically assimilated through natural selection, and become innate discrimination or mate preferences (Irwin and Price 1999; Magurran and Ramnarine 2005).

Here, we report a field experiment on two species of damselflies, *Calopteryx virgo* and *C. splendens*, a pair of species that hybridize in nature (Tynkkynen et al. 2008). Males of both species often court and try to mate with heterospecific females, and one potential explanation for matings between *C. virgo* and *C. splendens* is coercive behavior of males (Svensson et al. 2007; Tynkkynen et al. 2008). In the study, we presented females of both species to allopatric *C. virgo* males in repeated presentation trials, and observed the propensity of the males to mate with the females. With this experiment, we explored the possibility that learning may enhance species recognition through learning. Males could learn to associate their mating preference to conspecific females, and/or learn to discriminate heterospecific females. Because wild allopatric population was used as a study system, it is likely that *C. virgo* males had met conspecific females already, meaning that some associative learning could have happened in our study system before conducting the study. However, here our aim was to determine whether mature *C. virgo* males can still learn to discriminate heterospecific females or learn to associate with conspecific females during repeated experimental presentations of the heterospecific or conspecific females. Before that we tested whether allopatric *C. virgo* males, which have not encountered heterospecific females of the congener *C. splendens*, show initial discrimination against the heterospecific females, although the origin of possible discrimination (due to innate species recognition or associate learning) cannot be separated without using naïve males as study subjects.

## Materials and Methods

### Study species and hybridization

**Figure 1:**
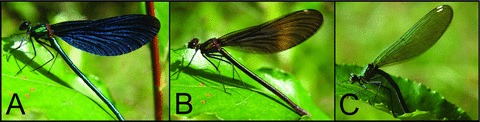
The study species. (A) *Calopteryx virgo* male and (B) female, and (C) *C. splendens* female. Note that the scale of the photographs differs. Photographs by Kaisa J. Raatikainen.

*C. virgo* L. 1758 and *C. splendens* Harris 1780 (Odonata: Calopterygidae) are two damselfly species, the distribution range of which overlaps widely across Europe (Askew 1988). In Finland, *C. splendens* is almost always sympatric with *C. virgo*, but allopatric *C. virgo* populations are quite common (seeTynkkynen et al. 2004). The two species have similar ecology, and they phenotypically resemble each other. Males are metallic blue, but females are either brownish (*C. virgo*) or greenish (*C. splendens*) (Fig. 1; Askew 1988). Males defend territories in rivers and streams, consisting of floating vegetation that females use as oviposition substrate. Females come to river habitats when they are ready to mate and lay eggs, and they do short flights to assess males and the oviposition sites (Hooper and Siva–Jothy 1997). Males display to females by presenting special courtship flight, which is easy to observe because of the high wing beating frequency (Pajunen 1966; Corbet 1999).

Hybrids between *C. splendens* and *C. virgo* occur in nature. However, the frequency of hybrids is low (0.13% of adults), compared to the observed frequency of heterospecific matings in the field (2.3% of matings;Tynkkynen et al. 2008; Kuitunen et al. 2011). This order of magnitude difference suggests that heterospecific matings may reduce the parental fitness. It may be that the viability of the hybrid offspring is lower compared to the pure bred individuals or females are mating with conspecific males after mating with the wrong species to reduce production of unfit offspring (seeSvensson et al. 2007; Tynkkynen et al. 2008). In the studies with *C. virgo*, it has been observed that males do not always discriminate against *C. splendens* females indicating that species recognition is not perfect (Svensson et al. 2007; Tynkkynen et al. 2008; Wellenreuther et al. 2010a). Indeed, when females of *C. splendens* were experimentally presented to territorial *C. virgo* males, 22% of the males court them, suggesting that *C. virgo* males have relatively poor species discrimination (Tynkkynen et al. 2008).

### Experimental setup

The study was performed between first and 17th of July 2006 on a 95-m section of the stream Tampinpuro in Central Finland (62°12′N, 25°3′E). In this stream, *C. virgo* is allopatric, meaning that we have not observed a single *C. splendens* individual in the population during three field seasons, and no nearby *C. splendens* populations are known. Thus, it is likely that mature *C. virgo* males in this population have had no previous encounters with *C. splendens* females but the males are likely to have previous experience with females of their own species. In the study section, most of the males were caught with butterfly nets and marked uniquely on their rear wings with a silver-marking pen (Artline® EK-999XF, Shachihata, Japan). This was done to ensure that we only present one set of females to each of the males once they had established a territory.

We performed an experiment in which we presented seven successive females (trials) for territorial *C. virgo* males, and observed the males’ propensity to mate. Territorial males were targets because we wanted to avoid possible variation that reproductive tactic can cause to male reactions (Tynkkynen et al. 2009). The propensity of the males to mate was determined by observing male behavior (see below) while moving mature, live females toward the males with the aid of a rod and fishing line (see Tynkkynen et al. 2008 and 2009 for more details of this technique). Females were tried to keep continuously flying to avoid them to perform rejection signals, which they can perform when perched (e.g., Pajunen 1966). Prior to the experiment, males were divided randomly into two groups: conspecific group and heterospecific group. In the conspecific group, five separate conspecific *C. virgo* females were presented to each of the 16 *C. virgo* males of conspecific group. In the heterospecific group, five separate heterospecific *C. splendens* females were presented to each of the 15 *C. virgo* males of heterospecific group. In both, the conspecific and heterospecific group, the sixth presentation consisted of presenting a *C. splendens* female to the focal *C. virgo* males (i.e., in the heterospecific group, *C. virgo* males actually met six *C. splendens* females successively). Finally, in the seventh presentation, *C. virgo* females were presented to the focal *C. virgo* males. The whole set of seven females was presented to males within 35 ± 26 min (mean ± SD; range 13–140 min). This relatively fast presentation time excludes the possibility to the time of a day-effect in male reactions.

Females that were used in the experiment were caught from a sympatric population ca. 60 km east from the study site (river Hohonjoki 62°15′N, 26°16′E). *Calopteryx* damselflies may prefer local familiar individuals as mates (Svensson et al. 2006; Wellenreuther et al. 2010b), and thus to avoid any familiarity bias, females of both species were caught from this population. The complete set of the seven individual females was presented for a given focal male during one day. Each of the females was presented up to two times but to separate males to decrease the number of females needed for the study (*N*= 129). All females that were in good condition after the experiment were marked with a silver-marking pen and released back to their own populations.

Male propensity to mate with a female was determined from his behavior during the female presentations. The behavior toward the females was ranked into seven categories of increasing propensity to mate: (1) an aggressive reaction (e.g., an aggressive attack), (2) no reaction (e.g., a male stays perched), (3) a nonaggressive reaction (e.g., an evasive movement), (4) some sexual interest (e.g., flying around a female without attacking or courting her), (5) courting less than 5 sec, (6) courting more than 5 sec, and (7) attempted tandem or a tandem. Each presentation lasted 20 sec.

### Statistical analyses and hypotheses

To determine whether *C. virgo* males show initial discrimination against *C. splendens* females, we compared the reactions of males to females between the conspecific and the heterospecific group during the first presentation trial. Learning of males was determined with two comparisons. First, we base our analysis on the assumption that learning takes place if male mating propensity changes during the female presentation trials. If there are differences between the first trial and from the second to the fifth trial in the conspecific group, it is indicative of learning to associate mating efforts to females of males’ own species. On the other hand, if there are differences between the first trial and from the second to the sixth trial in the heterospecific group, it is indicative of learning to strengthen species discrimination.

Second, the sixth and the seventh presentation trials were also planned to compare learning to discriminate against heterospecific or associate to conspecifics females: during the sixth trial, *C. virgo* males should discriminate heterospecific females more in the heterospecific than in the conspecific group if learning to the stronger species discrimination has occurred. During the seventh trial, *C. virgo* males should be more interested in the females of their own species in the conspecific than in the heterospecific group if association to conspecifics females has occurred. However, these comparisons can show learning only if males have not started to discriminate heterospecifics (conspecific group) or learnt to increase their preference to conspecifics (heterospecific group) as a by-product of the treatments during preceding presentation trials.

The data obtained from the learning experiment are categorical, meaning that we cannot infer parametric analysis with confidence (Zar 2010, see pp. 192, 249–250). Thus, the data were analyzed by using nonparametric tests. However, to get more confidence for results, we also reanalyzed five (conspecific group) or six (heterospecific group) first presentation trials with corresponding parametric analysis (repeated measures ANOVA). These results supported the results obtained from nonparametric tests (results not shown). Statistical testing was performed with SPSS (versions 14.0 and 16.0) and all the tests were two-tailed.

## Results

**Figure 2:**
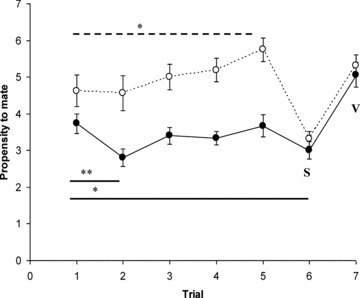
The propensity of *C. virgo* males to mate. In the conspecific group (dashed line and open circles), conspecific females were presented to *C. virgo* males during the first five trials. In the heterospecific group (solid line with filled circles), the first six females were heterospecifics (*C. splendens*). During the sixth presentation trial, males in both groups met *C. splendens* females (indicated by S), and during the seventh trial conspecific females were presented (indicated by V). Bars represent mean ± 1 SE. Horizontal lines indicate statistically significant differences between the first and from the second to the fifth (the conspecific group, dashed line) or to the sixth presentation trial (the heterospecific group, solid line). The linear increase of male mating propensity within the conspecific group was also detected with parametric statistical tests (results not reported here; see Materials and Methods section). Nonsignificant results not shown, *P < 0.05, **P < 0.01.

Our first aim was to determine whether the *C. virgo* males showed initial discrimination against the heterospecific females. To test this, the male reactions in the conspecific and in the heterospecific group during the first trial were compared. There was no significant difference between the groups, although there was a tendency for the conspecific group to show greater propensity to mate (Mann–Whitney U, *U*= 79.50, *N*_heterospecific group_= 15; *N*_conspecific group_= 16; *P*= 0.094). However, if males were divided for those that courted females (reactions 5–7) and those that did not court (reactions 1–4), conspecific females were more often courted than heterospecifics (62.5% vs. 13.3% of conspecific and heterospecific females were courted, respectively; χ^2^= 7.89, df = 1, *P*= 0.005). During all other trials, the difference in reactions was significant between the conspecific and the heterospecific group (for all comparisons: *U*≥ 30.5, *P*≤ 0.006;Fig. 2).

Our second aim was to determine whether *C. virgo* males learn to associate with conspecific females or learn to discriminate heterospecific females. This was done by examining whether changes occur in male reactions during five (conspecific group) or six (heterospecific group) first presentation trials. Within the first five or six trials, there were differences in the male propensity to mate within the conspecific (Friedman, χ*^2^*= 10.09, df = 4, *N*= 16, *P*= 0.039) and within the heterospecific group (Friedman, χ^2^= 14.50, df = 5, *N*= 15, *P*= 0.017;Fig. 2), respectively. In the conspecific group, testing whether there occurs association to conspecific females, males had a greater propensity to mate with conspecific females during the fifth trial than during the first one (Wilcoxon, *Z*=−2.30, *P*= 0.022), but there were no differences between the first and the other presentations (for all pairs: Z < −1.19, *P* > 0.24). Within the heterospecific group, testing the learning for species discrimination, males had a lower propensity to mate with conspecific females during the second and the sixth trial than the first one (*Z*=−2.80, *P*= 0.005 and *Z*=−2.33, *P*= 0.020, respectively), but there were no differences between the first and from the third to the fifth presentations (for all pairs: *Z* < −1.41, *P* > 0.16; Fig. 2). These results from the nonparametric analyses confirm those ones from parametric analyses.

Finally, we tested whether there was a difference between the conspecific and the heterospecific group in their response to females during the sixth and the seventh presentation trials, when males from both groups encountered heterospecific *C. splendens* and conspecific *C. virgo* females, respectively. During the sixth trial, there was no difference between the males of the conspecific and the heterospecific group (Mann–Whitney U, *U*= 96.50, *P*= 0.31). Similarly, during the seventh trial, there was no difference between the males of the conspecific and the heterospecific group (*U*= 108.50, *P*= 0.63).

## Discussion

Our results suggest that *C. virgo* males have at least tendency for initial preference for conspecific females, although the result was not significant (*P*= 0.094). However, if males were divided for those that courted females (reactions 5–7) and those that did not court (reactions 1–4), conspecific females were more often courted than heterospecifics. This suggests that nonsignificant difference during the first presentation trial between experimental groups might also be caused by more accurate division of male reactions in our study. Initial discrimination has been reported also from other allopatric populations of *C. virgo* (Wellenreuther et al. 2010a). During all other presentation trials than the first one, males had stronger propensity to mate with a female in the conspecific group than in the heterospecifc group. This may be due to that *C. virgo* males of either or both experimental groups started to adjust their mate recognition fast to the females of their own species. It is likely that males have already encountered females of their own species, and thus association for *C. virgo* females can already have taken place. It should be noted here that our study was not designed to separate innate and association learning because naïve males were not used.

Although the learning to conspecific females might already have occurred before our experiment, this does not exclude the possibility that further associative learning may occur in the mature individuals. Indeed, signs of this kind of learning were observed in our study: *C. virgo* males in the conspecific group seemed to increase their propensity to mate with conspecific females during the first five presentation trials until there was statistical difference between the first and fifth presentation trials (seeFig. 2). In addition, the results suggest that the learning to discriminate heterospecific females might have some influence on *C. virgo* males, because they were more discriminating against *C. splendens* females during the second and the sixth presentation trials than during the first. However, there was no difference between the first trial and all the other trials with *C. splendens* females in male reactions. Thus, it is not so clear whether discriminative learning against heterospecific females occurs in *C. virgo* males or not.

There are several alternatives for learning, which could explain why *C. virgo* males increased their propensity to mate with the conspecific females during the repeated trials. For instance, repeated encounters with the conspecific females on the territory of the male might function as a stimulus, which increases the male's physiological state to mate. In addition, although males increased their propensity to mate with *C. virgo* females, it seems that this effect was not persistent. This is because there was no difference between the conspecific and the heterospecific group in the propensity of the males to mate with a female during the seventh trial, when the males of both the conspecific and the heterospecific groups encountered a conspecific female. If we think that learning was the reason why *C. virgo* males increased their propensity to mate with conspecifics, the males of the conspecific group should still have greater propensity to mate with conspecific females than the males of the heterospecific group. However, the presence of conspecific females at the river may have enhanced learning to conspecific females also in the heterospecific group, leading to nonsignificant difference between the heterospecific and the conspecific group.

During the sixth and the seventh presentation trials, when heterospecific or conspecific females were presented, respectively, we tried to tease out the relative importance of the different forms of learning. This would have been possible if a clear difference between the conspecific and the heterospecific group had appeared. However, this was not the case: in both cases, the conspecific males were as discriminating as the males in the heterospecific group. This suggests that initial species recognition was at a level that cannot be enhanced greatly by learning. Alternatively, it can be that learning to associate mating efforts to conspecific females increases learning to discriminate as a by-product, and vice versa, leading to uncertain conclusions from our experimental setup concerning the sixth and the seventh presentation trials. In addition, it should be kept in mind that the local free-flying *C. virgo* females are affecting reactions of the males, at least they have potential to increase learning of males to their own species.

Our experiment could be criticized because we had no clear negative or positive reward to the males for their behavior, which may cause learning to be less effective. However, the behavior of the females in our experiment is likely to be close to that in natural settings, since most males are rejected even by conspecific females (K. Kuitunen, pers. obs.). In addition, it has been observed in male fruitflies (*Drosophila pseudoobscura*) that learning to avoid heterospecific females can occur even in cases where they do not get matings from conspecifics (Dukas 2009).

For each male, our experiment was completed on average within 35 min, which might appear as a too short time frame for males to learn. However, males should be fast learners because their short life span of about four to six days (see Svensson et al. 2006; Svensson and Friberg 2007; Tynkkynen et al. 2009). Because of such a short life span, learning should occur relatively fast to be efficient to prevent males from wasting courting and mating efforts with females that provide little or even negative fitness rewards. It could also be that males should react fast to any female irrespective of their species just in order to avoid missing of potential conspecific mates (seeCorbet 1999; Tynkkynen et al. 2009). Indeed, when individuals of the other sex, or females in this case (Svensson et al. 2007), are good in species discrimination, selection to avoid matings with heterospecifics may be relaxed in the other sex (Parker and Partridge 1998; Peterson et al. 2005). Similarly, it may be that when one sex is good in species recognition, the other could be relaxed from the pressure to learn.

Svensson et al. (2010) have studied learning for species discrimination in *C. splendens* females. In their study, *C. virgo* and *C. splendens* males were presented to initially sexually naïve *C. splendens* females after exposing them to males of either species in cages. Their results suggest that learning against *C. virgo* males takes place in *C. splendens* females, but females do not increase their mating propensity to conspecific males after the exposure to conspecifics (Svensson et al. 2010). In our study with free-living *C. virgo* males, it seems that the main conclusion is opposite for the findings ofSvensson et al. (2010), that is, *C. virgo* males started to associate their mating preference to conspecific females. These contrasting findings, although with individuals of different species, might suggest that preference for mates of their own species may evolve through different mechanisms in males and females.

As a conclusion, we suggest that learning to discriminate between con- and heterospecifics (Svensson et al. 2010) as well as learning to prefer conspecifics might be important in species discrimination in damselflies. The former type of learning can (by definition) only take place in sympatry, where both species regularly encounter each other (Price 2007), but the latter might operate also in allopatric populations. Associative learning may be involved in mate recognition, and might be reflected as a significant preference for conspecific females, even if heterospecific females have never been encountered before. However, such learning might be important and it could develop during the course of the life span of individual males and females, as they gradually encounter and learn to recognize potential mates among conspecifics. Thus, learning might play a role in enhancement of premating reproductive isolation also when individuals are not naïve but have already encountered potential conspecific mates.
